# Effects of high-intensity interval training on body morphology and mental health in obese female college students

**DOI:** 10.3389/fmed.2026.1832286

**Published:** 2026-06-17

**Authors:** Kerui Zhu

**Affiliations:** School of Physical Education, Guangzhou College of Commerce, Guangzhou, Guangdong, China

**Keywords:** body morphology, female college students, high-intensity interval training, mental health, obesity, physical fitness, quality of life

## Abstract

**Aim:**

To investigate the impacts of high-intensity interval training (HIIT) on body morphology along with mental health in obese female college students.

**Methods:**

One hundred and twenty obese female college students admitted to our university from October 2022 to October 2025 were included and randomly divided into control group and study group. The former implemented routine health education without any structured exercise intervention, while the latter underwent a 12-week HIIT intervention (three sessions per week, 30 min per session). Comparisons included Body morphology indicators, mental health indicators, biochemical indicators, sleep quality along with quality of life.

**Results:**

Following the intervention, compared to the control group, the study group demonstrated significant improvements across multiple metrics. Specifically, the study group exhibited a lower body mass index, reduced body fat percentage, decreased waist circumference, and a lower waist-to-hip ratio (*P* < 0.05). In terms of mental health, the study group had lower scores on the PHQ-9 for depression and the GAD-7 for anxiety, while showing higher scores on the SES for self-esteem (*P* < 0.05). Physical fitness improvements were also notable, with the study group displaying higher VO_2_max and vital capacity (*P* < 0.05). Additionally, the study group showed better lipid profiles, lower fasting blood glucose levels, and improved sleep quality as indicated by lower PSQI scores (*P* < 0.05). Finally, the study group scored higher on all dimensions of the SF-36 quality of life scale (*P* < 0.05).

**Conclusion:**

HIIT intervention can effectively improve body morphology, mental health, physical fitness, biochemical indicators, sleep quality, and quality of life in obese female college students.

## Introduction

Obesity has become a major global public health concern, with substantial effects on both physical and mental wellbeing (1). Based on the World Health Organization, the occurrence rate of obesity has nearly increased threefold since 1975. Approximately 39% adults aged 18 and above are overweight, while 13% are classified as obese (2). Among college students, the rates of overweight and obesity have been rising, driven by sedentary lifestyles, unhealthy dietary patterns, and low levels of physical activity (3). Female college students are especially prone to weight gain during college, which may lead to long-term adverse effects on their health and quality of life (4).

Recent evidence suggests that the burden of overweight and obesity has continued to rise beyond the historical benchmark cited in earlier reports. According to the World Health Organization, 43% of adults worldwide were overweight and 16% were living with obesity in 2022 (5). In Asia (6), urbanization has been consistently associated with higher obesity risk, particularly in rapidly developing urban environments. Among university students, overweight and obesity remain important health concerns, and abnormal BMI has been associated with poorer physical fitness performance (7, 8). These data highlight the importance of targeted exercise interventions for female college students in urban Asian settings.

Obesity is linked to a wide range of physical disorders, containing cardiovascular diseases, type 2 diabetes, hypertension, along with dyslipidemia (9). In addition to physical health burdens, obesity also exerts substantial negative effects on mental health. Studies have indicated that individuals with obesity exhibit higher depression, anxiety, as well as lower self-esteem relative to normal-weight individuals (10). The association between obesity and mental health is bidirectional and complex: psychological distress may trigger maladaptive coping behaviors containing emotional eating and physical inactivity, which further worsen obesity (11).

High-intensity interval training (HIIT) has attracted significant attention because of its potential in improving cardiovascular fitness as well as decreasing body fat within a comparatively short period (12). HIIT involves alternating short bursts of intense physical activity with intervals of rest or low-intensity recovery (13), making it highly suitable for young people with busy schedules and limited time for conventional exercise. Research has shown that HIIT can trigger physiological changes akin to those produced by conventional, prolonged aerobic exercises, but does so in a manner that is much more time-efficient (14).

Previous studies have documented the positive impacts of HIIT on multiple health outcomes among individuals who are overweight or obese. According to Song et al.'s study, HIIT notably enhanced both body composition and inhibitory control among overweight college students (15). In another study, Martland et al. found that HIIT resulted in moderate improvements in mental wellbeing, depressive symptoms, as well as perceived stress (16).

Although accumulating evidence supports the physical health benefits of HIIT, limited research has comprehensively examined its simultaneous effects on body morphology and multiple domains of mental health among obese female college students. Additionally, the optimal duration and intensity of HIIT for this population remain unclear. The present study comprehensively assessed the effects of a 12-week HIIT intervention on body morphology, mental health, physical fitness, biochemical markers, sleep quality, quality of life, and intervention adherence.

## Methods

### Study design

A total of 134 obese female college students admitted from October 2022 to October 2025 were chosen as research participants. Inclusion criteria: (1) BMI ≥ 28 kg/m^2^ based on Chinese obesity criteria; (2) age 18–25 years; (3) no regular exercise habits (exercise < 2 times per week, < 30 minutes per session); (4) no participation in any structured weight loss programs in the past 3 months. Exclusion criteria: (1) Secondary obesity due to endocrine disorders or medications; (2) cardiovascular diseases, respiratory diseases, or musculoskeletal disorders contraindicating high-intensity exercise; (3) a known history of psychiatric disorders, severe psychological conditions requiring medication, or other psychological conditions identified through medical history, self-report, and pre-enrollment interview that could interfere with study participation; (4) pregnancy or lactation; (5) smoking or excessive alcohol consumption; (6) incomplete clinical data. By employing a random number table approach, the patients were evenly allocated into the control group and the study group, each comprising 67 participants. This research received approval from the university's ethics committee. All individuals involved provided their written informed consent. Before enrollment, all participants underwent routine pre-study interview-based screening, including review of medical history, current medication use, and self-reported psychological status, to exclude individuals with previously diagnosed psychiatric disorders or severe mental health conditions.

### Interventions

The control group received routine health education, including information on a healthy diet and the benefits of physical activity, but did not participate in any structured exercise intervention during the 12-week study period. Participants in this group were asked to maintain their usual lifestyle and dietary habits throughout the study.

The study group underwent a 12-week high-intensity interval training (HIIT) intervention. Each session lasted 30 min and was performed three times per week (Monday, Wednesday, and Friday), for a total of 36 sessions. Each training session consisted of a 5-min warm-up, a 20-min HIIT phase, and a 5-min cool-down. The warm-up included light aerobic activity such as jogging or dynamic stretching, whereas the cool-down consisted of static stretching exercises.

During the main HIIT phase, participants completed eight exercises, including jumping jacks, squats, push-ups, mountain climbers, high knees, burpees, lunges, and plank jacks. Each exercise was performed for 40 s at maximal intensity, followed by 20 s of rest. Two rounds of the 8-exercise circuit were completed, with a 1-min rest interval between rounds.

Exercise intensity was maintained at 85%−95% of the estimated maximum heart rate (HRmax = 220–age). Heart rate was monitored using Polar heart rate monitors during training sessions. The rating of perceived exertion (RPE) was assessed using the Borg 6–20 scale, with a target RPE of 15–18.

All exercise sessions were supervised by certified fitness trainers. Participants were instructed to report any discomfort or adverse symptoms immediately. Blood pressure was measured before and after each session during the first week and once weekly thereafter. Attendance was recorded throughout the intervention period. Participants who missed more than 20% of sessions (≥7 sessions) were considered non-adherent and were excluded from the final analysis.

### Observation indicators

Body morphology indicators, including body mass index (BMI), body fat percentage, waist circumference, hip circumference, and waist-to-hip ratio, were measured before the intervention and again after 12 weeks.

Mental health indicators were assessed before the intervention and after 12 weeks using standardized instruments. All instruments have demonstrated good internal consistency in Chinese college populations: Cronbach's α for PHQ-9 = 0.86–0.89, GAD-7 = 0.87–0.91, SES = 0.81–0.85, PSQI = 0.83, and SF-36 subscales ranged from 0.78 to 0.90. Depressive symptoms were assessed using the Patient Health Questionnaire-9 (PHQ-9) (17), and anxiety symptoms were evaluated using the Generalized Anxiety Disorder-7 (GAD-7) (18). These instruments were used to assess symptom severity during the study rather than as obesity-specific pre-recruitment screening tools. Self-esteem was measured using the Rosenberg Self-Esteem Scale (SES) (19), where higher scores indicate higher self-esteem.

Physical fitness indicators were also measured before and after the intervention. Maximal oxygen uptake (VO_2_max) was determined using a graded exercise test on a treadmill with gas exchange analysis, and vital capacity was measured using a spirometer.

For biochemical assessment, fasting venous blood samples were collected before the intervention and after 12 weeks to determine fasting blood glucose, triglycerides (TG), total cholesterol (TC), high-density lipoprotein cholesterol (HDL-C), and low-density lipoprotein cholesterol (LDL-C).

Sleep quality was assessed before and after the intervention using the Pittsburgh Sleep Quality Index (PSQI) (20). Quality of life was evaluated using the 36-Item Short-Form Health Survey (SF-36) (21), which includes eight dimensions, with higher scores indicating better quality of life.

### Statistical analysis

Continuous variables were expressed as mean ± standard deviation (*x* ± *s*), and between-group comparisons were performed using the independent-samples *t*-test. Categorical variables were expressed as numbers and percentages (%), and between-group comparisons were performed using the χ^2^ test. A *P* value of < 0.05 was considered statistically significant.

## Results

### General information

A total of 134 obese female college students were enrolled, with 67 in each group. In the study group, 60 participants completed the full intervention (90.0% adherence), and seven were excluded due to non-adherence. In the control group, 60 participants completed full intervention assessments, and seven were lost to follow-up. Final analysis included 120 participants. No discrepancies were reported in baseline characteristics of both groups (*P* > 0.05, Table 1), indicating comparability.

**Table 1:** Comparison of general information between the two groups (*x* ± *s*).

General information	Control group (*n* = 60)	Study group (*n* = 60)	*t*/χ^2^ value	*P* value
Age (years)	20.56 ± 1.67	20.78 ± 1.71	0.672	0.503
Height (cm)	163.45 ± 4.89	164.12 ± 5.03	0.698	0.487
Weight (kg)	75.67 ± 6.34	76.23 ± 6.45	0.452	0.652
BMI (kg/m^2^)	28.34 ± 2.21	28.31 ± 2.18	0.071	0.944
Body fat percentage (%)	34.56 ± 3.45	34.48 ± 3.51	0.119	0.906

### Body morphology indicators

**Figure 1:**
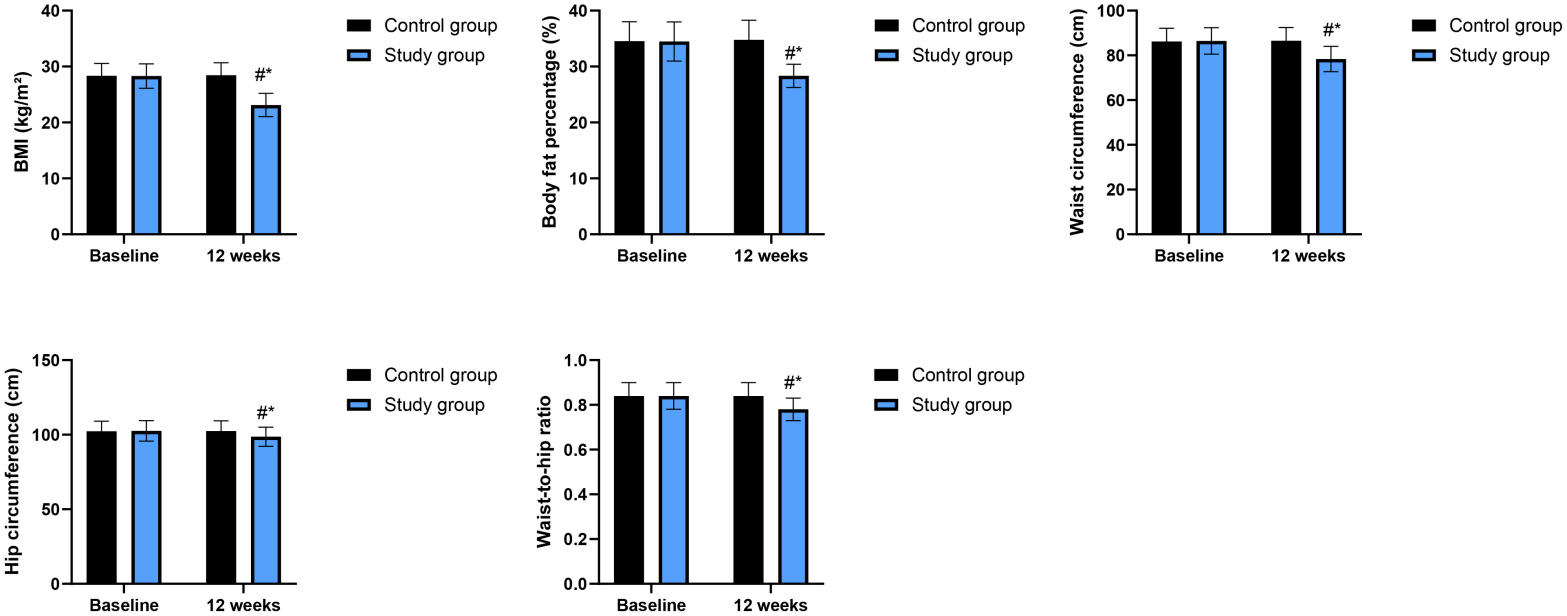
Comparison of body morphology indicators between the two groups before and after intervention. Compared with before intervention within the same group, *P < 0.05; compared with the control group after intervention, ^#^*P* < 0.05.

Before intervention, no differences were reported in body morphology indicators between both groups (*P* > 0.05). Following intervention, the study group showed significant reductions in all body morphology indicators relative to baseline, while the control group exhibited no significant alterations. The study group had significantly lower BMI, body fat percentage, waist circumference, as well as waist-to-hip ratio relative to the control group following intervention (*P* < 0.05, Figure 1).

### Mental health indicators

**Figure 2:**
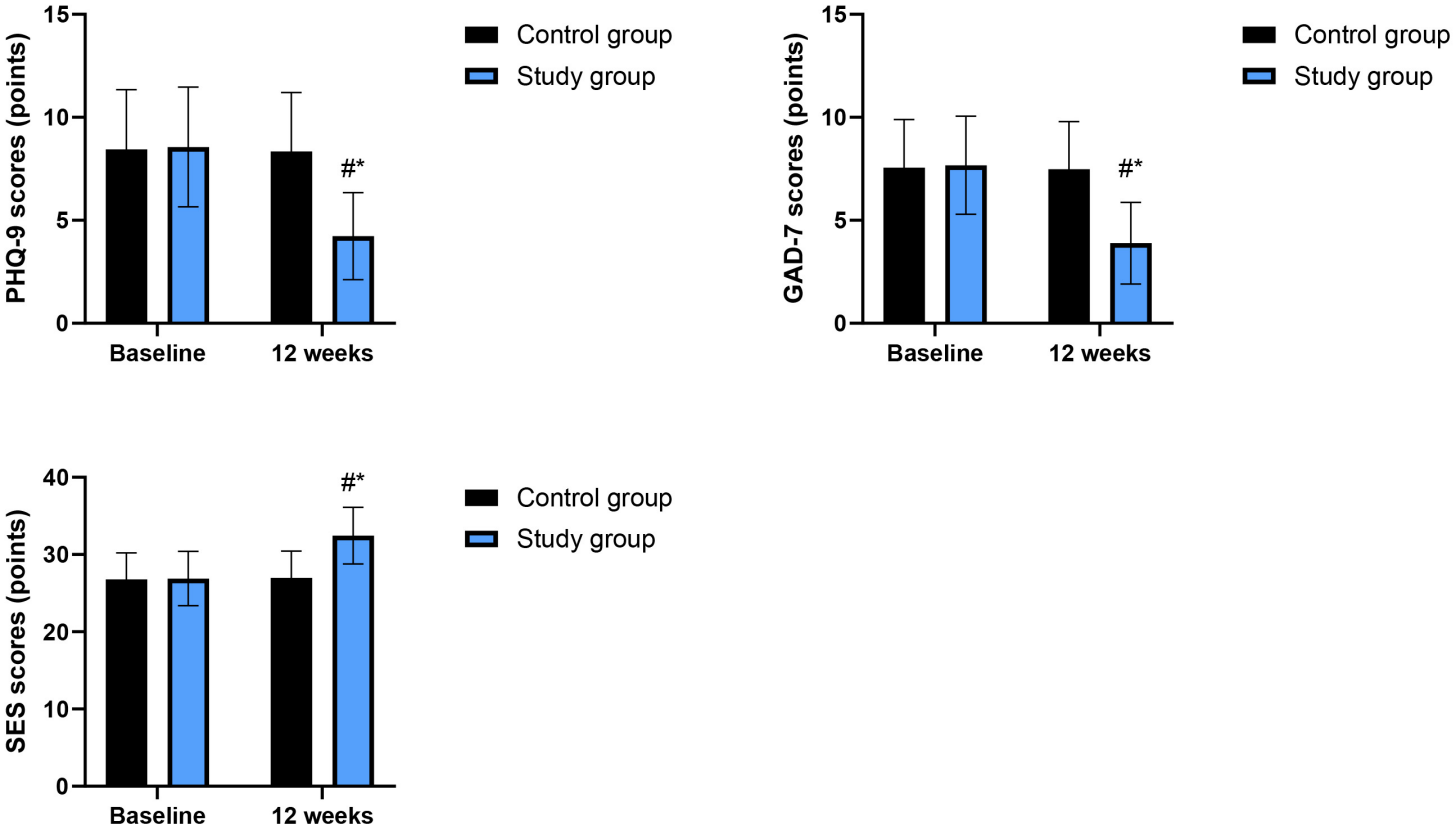
Comparison of mental health indicators between the two groups before and after intervention. Compared with before intervention within the same group, *P < 0.05; Compared with the control group after intervention, ^#^*P* < 0.05.

Before intervention, no differences were reported in mental health indicators between both groups (*P* > 0.05). Following intervention, the study group showed obvious improvements in all mental health indicators relative to baseline, while the control group exhibited no significant alterations. The study group had significantly lower PHQ-9 scores (depression) and GAD-7 scores (anxiety), and significantly higher SES scores (self-esteem) relative to the control group following intervention (*P* < 0.05, Figure 2).

### Physical fitness indicators

**Figure 3:**
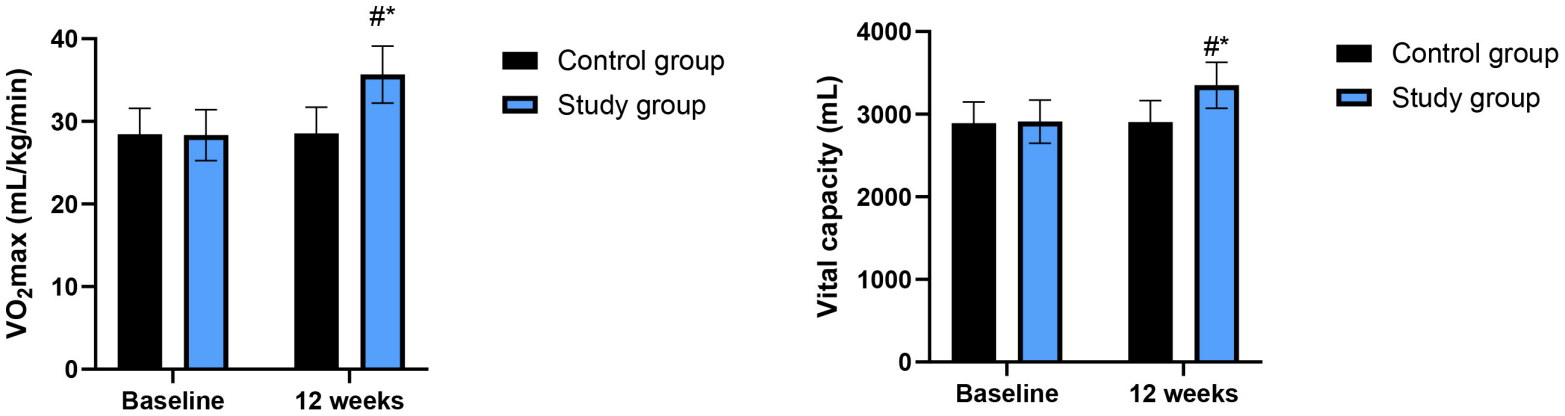
Comparison of physical fitness indicators between the two groups before and after intervention. Compared with before intervention within the same group, *P < 0.05; Compared with the control group after intervention, ^#^*P* < 0.05.

Before intervention, no differences were reported in physical fitness indicators between both groups (*P* > 0.05). Following intervention, the study group demonstrated elevations in VO_2_max and vital capacity relative to baseline, while the control group exhibited no significant changes. The study group exhibited higher VO_2_max and vital capacity relative to the control group following intervention (*P* < 0.05, Figure 3).

### Biochemical indicators

**Figure 4:**
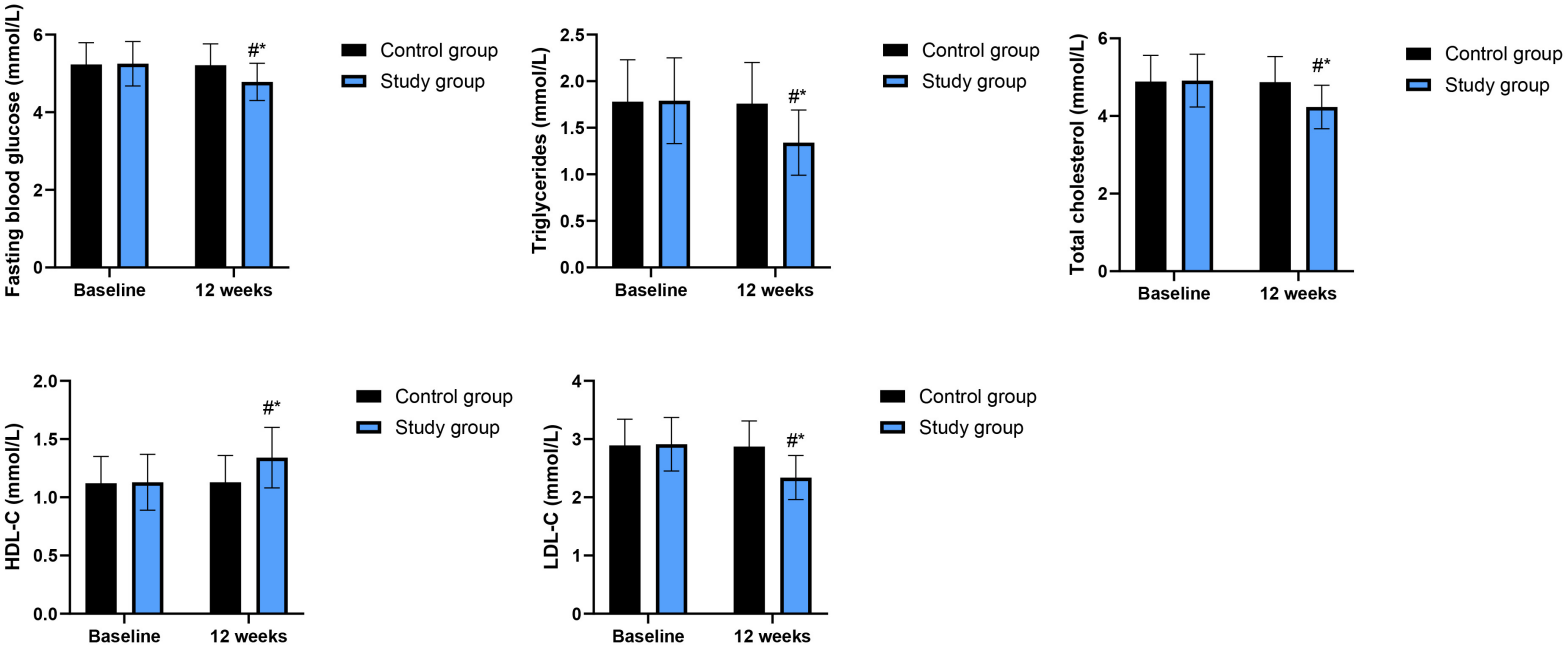
Comparison of biochemical indicators between the two groups before and after intervention. Compared with before intervention within the same group, *P < 0.05; Compared with the control group after intervention, ^#^*P* < 0.05.

Before intervention, no differences were reported in biochemical indicators between both groups (*P* > 0.05). Following intervention, the study group exhibited significant improvements in fasting blood glucose along with lipid profile relative to baseline, while the control group showed no significant alterations. The study group had significantly lower fasting blood glucose, triglycerides, total cholesterol, and LDL-C, along with significantly higher HDL-C relative to the control group following intervention (*P* < 0.05, Figure 4).

### Sleep quality

**Figure 5:**
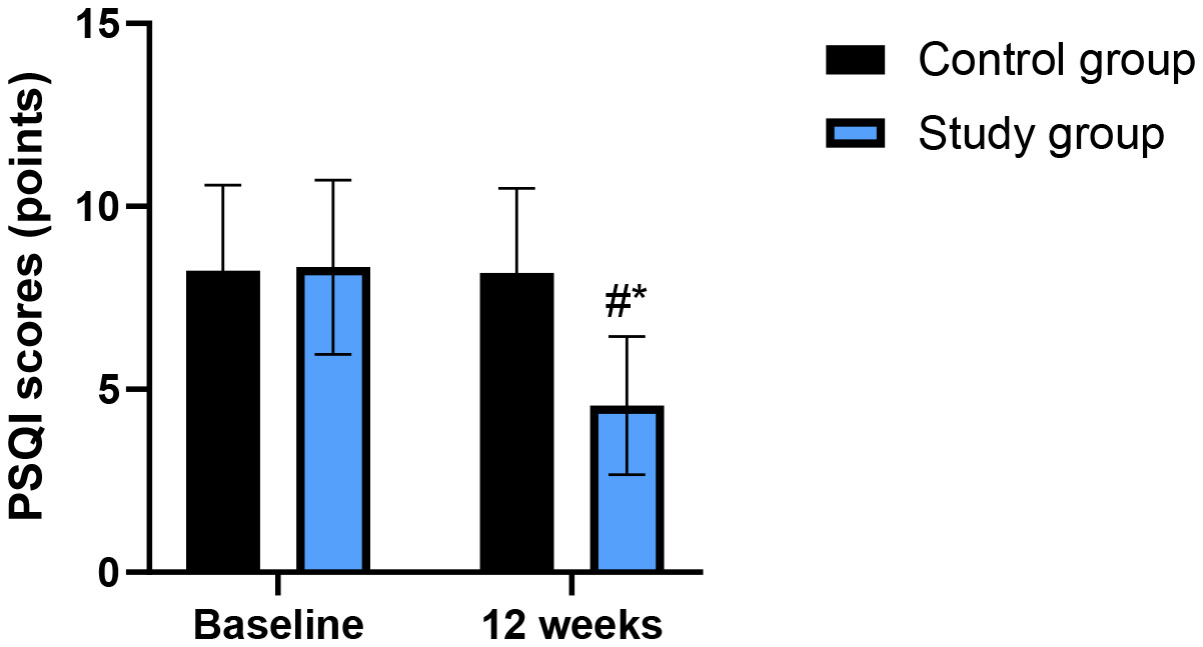
Comparison of PSQI scores between the two groups before and after intervention. Compared with before intervention within the same group, *P < 0.05; Compared with the control group after intervention, ^#^*P* < 0.05.

Before intervention, no discrepancies were reported in PSQI scores between both groups (*P* > 0.05). Following intervention, the study group showed significant improvement in sleep quality relative to baseline, while the control group presented no notable change. The study group had significantly lower PSQI scores relative to the control group following intervention (*P* < 0.05, Figure 5).

### Quality of life scores

**Figure 6:**
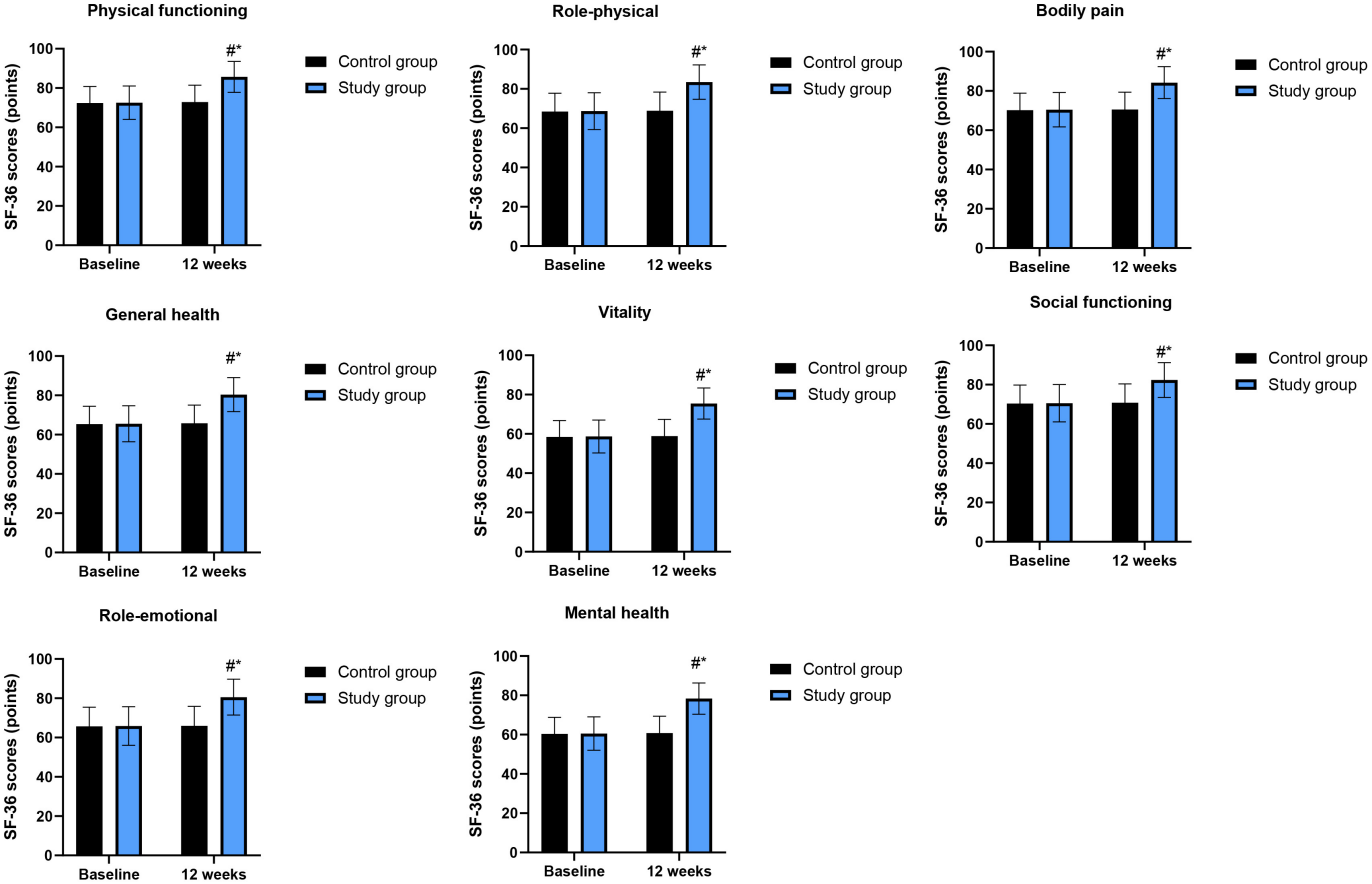
Comparison of SF-36 scores between the two groups before and after intervention. Compared with before intervention within the same group, *P < 0.05; Compared with the control group after intervention, ^#^*P* < 0.05.

Before intervention, no discrepancies were reported in SF-36 scores between both groups (*P* > 0.05). Following intervention, the study group showed significant elevations in all dimensions of quality of life relative to baseline, while the control group exhibited no significant alterations. The study group exhibited significantly higher SF-36 scores in all dimensions relative to the control group following intervention (*P* < 0.05, Figure 6).

## Discussion

HIIT has emerged as a time-efficient exercise modality for improving both physical along with mental health in various populations (22). This study comprehensively evaluated the impacts of a 12-week HIIT on body morphology and mental health in obese female college students.

This research demonstrated that HIIT significantly improved all body morphology indicators in obese female college students. The study group exhibited significant reductions in BMI, body fat percentage, waist circumference, as well as waist-to-hip ratio after intervention, with all changes being greater than those in the control group. Consistently, research conducted by Zuo et al. revealed that engaging in an 8-week HIIT program significantly enhanced BMI, lean body mass, and reduced body fat content among overweight female college students (23). Similarly, the study by Gaweł et al. also demonstrated that HIIT combined with dietary supplementation significantly improved body composition in in adults with overweight and obesity (24). The mechanisms underlying HIIT-induced improvements in body morphology are multifactorial. HIIT boosts energy expenditure not only during the workout but also afterward, thanks to excess post-exercise oxygen consumption (EPOC). The intense nature of HIIT encourages higher rates of fat oxidation while helping to maintain lean muscle mass (25). Additionally, HIIT has been demonstrated to enhance insulin sensitivity and optimize lipid metabolism, thereby aiding in the reduction of fat accumulation (26).

A significant finding of this study was the substantial improvement in mental health indicators following HIIT intervention. The study group exhibited significant decreases in depression scores and anxiety scores, and significant increases in self-esteem scores after intervention. Similarly, Zeng and Wang suggested that HIIT modestly improved depressive symptoms in patients with depression (27). The psychological benefits of HIIT can be attributed to several mechanisms. First, exercise stimulates the release of endorphins and neurotransmitters containing serotonin and dopamine, which improve mood and decrease symptoms of depression and anxiety (28). Second, the achievement of exercise goals and visible improvements in body composition can enhance self-efficacy and self-esteem. Third, the group exercise setting may provide social support and reduce feelings of isolation. Fourth, improved sleep quality, as observed in our study, can positively impact mental health (29).

The HIIT intervention significantly improved cardiorespiratory fitness in obese female college students. The study group showed significant increases in VO_2_max and vital capacity after intervention. These improvements are in accordance with previous literatures. Poon et al. reported significant elevation in VO_2_max and vital capacity after HIIT in obese middle-aged men (30). The improvement in cardiorespiratory fitness is a key benefit of HIIT. The high-intensity intervals provide a strong stimulus for cardiac adaptation, including increased stroke volume and cardiac output, while the recovery periods allow for sufficient rest to maintain high-intensity effort throughout the session. These adaptations translate to improved aerobic capacity and exercise tolerance.

The HIIT intervention significantly improved metabolic health indicators in obese female college students. The study group showed significant reductions in fasting blood glucose, triglycerides, total cholesterol, and LDL-C, long with elevation in HDL-C after intervention. Likewise, Cicek et al. reported that aerobic and HIIT improved glucose, insulin, HOMA-IR, LDL-C in overweight and obese women (31). The metabolic improvements observed can be attributed to several factors. HIIT increases insulin sensitivity along with glucose uptake by skeletal muscle, leading to improved glycemic control. The high-intensity nature of the exercise promotes greater fat oxidation and improves lipid metabolism. Additionally, reductions in body fat, particularly visceral fat, contribute to improved metabolic profile.

A notable finding of this research was the significant improvement in sleep quality following HIIT intervention. The study group showed significant reductions in PSQI scores after intervention, indicating better sleep quality. Consistently, it has been reported that HIIT can improve sleep quality and sleep efficiency of adults (32). The improvement of sleep quality may be the result of multiple mechanisms working together. Exercise promotes sleep by increasing body temperature, causing a decrease in temperature, alleviating anxiety and depression symptoms, and regulating the circadian rhythm. The relationship between the improvement of sleep quality and mental health is bidirectional. Good sleep helps to improve mood and reduce anxiety, while good mental health helps to improve sleep.

The HIIT intervention significantly improved all dimensions of quality of life scores in obese female college students. These comprehensive improvements reflect the multifaceted benefits of HIIT. Improved physical fitness and body composition enhance physical functioning and reduce bodily pain. Better mental health and self-esteem contribute to improved emotional wellbeing and social functioning. Increased energy levels and vitality improve participation in daily activities and social roles. The combination of these improvements leads to enhanced overall qualityr of life.

The findings of this research are largely consistent with previous research on HIIT in overweight and obese populations. Guo et al. demonstrated that HIIT exerts a positive impact on the body composition of adults who are overweight or obese (33). Hadjispyrou et al. reported that HIIT enhances mitochondrial-related indices in individuals who are overweight or obese (34). Fu et al. suggested that HIIT proves effective in enhancing psychological wellbeing as well as optimizing sleep patterns among female students who exhibit normal weight obesity (35). However, our study extends previous research in several ways. First, we comprehensively evaluated seven categories of outcomes, providing a more complete picture of HIIT's effects. Second, we included self-esteem as a mental health outcome, which has been less frequently studied in HIIT research. Third, we assessed quality of life using the SF-36, providing insights into how HIIT affects daily functioning and wellbeing. Fourth, we documented intervention adherence, providing practical information for program implementation.

Beyond its effects on body composition and physical fitness, exercise is increasingly regarded as a therapeutic and preventive strategy in modern health care (36). The Exercise is Medicine initiative advocates integrating physical activity assessment and promotion into routine clinical practice (37). In this context, our findings further support the view that structured exercise interventions such as HIIT may contribute not only to obesity management, but also to broader improvements in mental wellbeing, metabolic health, sleep quality, and quality of life in young adults (16, 38).

This research has some limitations. First, the sample was drawn from a single university, which may restrict generalizability to other populations. Second, the 12-week intervention period, while enough to observe significant changes, does not provide information on long-term maintenance of improvements. Third, we did not include a comparison group receiving moderate-intensity continuous training, which would allow direct comparison of the relative effectiveness of different exercise modalities. Fourth, dietary intake was not controlled or monitored, which may have influenced outcomes. Fifth, self-report measures of mental health, while validated, may be subject to reporting bias. Sixth, the dropout rate of 15% in the study group, while acceptable, may introduce some selection bias. Sixth, no formally programmed progression or variation of exercise movements was incorporated across the 12-week intervention, which may have influenced long-term adaptability and training responsiveness. Future studies should explore more dynamic HIIT protocols with structured progression. In addition, no obesity-specific psychological screening or quality-of-life instrument, such as ORWELL-97 or IWQOL-Lite, was used during recruitment, which may have limited the identification of obesity-related psychological burden and weight-specific quality-of-life impairment. Future studies should incorporate obesity-specific instruments together with general mental health scales to improve the comprehensiveness of participant characterization. Moreover, future research should include longer follow-up periods to assess maintenance of improvements, compare HIIT with other exercise modalities, control for dietary intake, and include objective measures of physical activity and sleep. Studies exploring the optimal HIIT protocol (intensity, duration, frequency) for this population are also needed.

## Conclusion

In summary, this study demonstrates that a 12-week HIIT intervention effectively improves body morphology, mental health, physical fitness, biochemical indicators, sleep quality, and quality of life in obese female college students. The high adherence rate (85.0%) suggests that HIIT is a feasible and acceptable intervention for this population. These findings highlight the potential of HIIT as a practical and time-efficient strategy for obesity management and mental health improvement in young adults. However, the results should be interpreted with caution due to the single-center design and relatively short intervention period, and further studies with larger samples and longer follow-up are warranted.

## Data Availability

The original contributions presented in the study are included in the article/supplementary material, further inquiries can be directed to the corresponding author.

## References

[1] Wang Y Zhao L Gao L Pan A Xue H. Health policy and public health implications of obesity in China. Lancet Diabetes Endocrinol. (2021) 9:446–61. doi: 10.1016/S2213-8587(21)00118-2 34097869

[2] Liu C Yuan YC Guo MN Xin Z Chen GJ Ding N . Rising incidence of obesity-related cancers among younger adults in China: a population-based analysis (2007-2021). Med. (2024) 5:1402-1412.e2. doi: 10.1016/j.medj.2024.07.012 39181132 PMC11560649

[3] Mondal S Basu C Alkhawaitri M Almamari I Albrwaney S Alhabsi T. Obesity among college students in Oman: implications for health and academic performance. BMC Public Health. (2025) 25:1111. doi: 10.1186/s12889-025-21946-7 40128737 PMC11931841

[4] Aryal V Ghimire D Kandel S Majumder A Manna S. Obesity among Medical Students of a Medical College: A Descriptive Cross-sectional Study. JNMA J Nepal Med Assoc. (2022) 60:943–6. doi: 10.31729/jnma.7519 36705181 PMC9795093

[5] World Health Organization. Obesity and Overweight. Geneva: World Health Organization (2025).

[6] Angkurawaranon C Jiraporncharoen W Chenthanakij B Doyle P Nitsch D. Urban environments and obesity in southeast Asia: a systematic review, meta-analysis and meta-regression. PLoS ONE. (2014) 9:e113547. doi: 10.1371/journal.pone.0113547 25426942 PMC4245122

[7] Peltzer K Pengpid S Samuels TA Özcan NK Mantilla C Rahamefy OH . Prevalence of overweight/obesity and its associated factors among university students from 22 countries. Int J Environ Res Public Health. (2014) 11:7425–41. doi: 10.3390/ijerph110707425 25050651 PMC4113885

[8] Guo T Shen S Yang S Yang F. The relationship between BMI and physical fitness among 7451 college freshmen: a cross-sectional study in Beijing, China. Front Physiol. (2024) 15:1435157. doi: 10.3389/fphys.2024.1435157 39473612 PMC11519527

[9] Muscogiuri G Verde L Sulu C Katsiki N Hassapidou M Frias-Toral E . Mediterranean diet and obesity-related disorders: what is the evidence? Curr Obes Rep. (2022) 11:287–304. doi: 10.1007/s13679-022-00481-1 36178601 PMC9729142

[10] Melamed OC Selby P Taylor VH. Mental health and obesity during the COVID-19 pandemic. Curr Obes Rep. (2022) 11:23–31. doi: 10.1007/s13679-021-00466-6 35254633 PMC8899440

[11] Kemp JVA Kumar V Saleem A Hashman G Hussain M Taylor VH. Examining associations between women's mental health and obesity. Psychiatr Clin North Am. (2023) 46:539–49. doi: 10.1016/j.psc.2023.04.009 37500249

[12] Coates AM Joyner MJ Little JP Jones AM Gibala MJ A. Perspective on high-intensity interval training for performance and health. Sports Med. (2023) 53:85–96. doi: 10.1007/s40279-023-01938-6 37804419 PMC10721680

[13] Wu ZJ Wang ZY Gao HE Zhou XF Li FH. Impact of high-intensity interval training on cardiorespiratory fitness, body composition, physical fitness, and metabolic parameters in older adults: a meta-analysis of randomized controlled trials. Exp Gerontol. (2021) 150:111345. doi: 10.1016/j.exger.2021.111345 33836261

[14] Atakan MM Li Y Koşar S N Turnagöl HH Yan X. Evidence-based effects of high-intensity interval training on exercise capacity and health: a review with historical perspective. Int J Environ Res Public Health. (2021) 18:7201. doi: 10.3390/ijerph18137201 34281138 PMC8294064

[15] Song X Cui X Su W Shang X Tao M Wang J . Comparative effects of high-intensity interval training and moderate-intensity continuous training on weight and metabolic health in college students with obesity. Sci Rep. (2024) 14:16558. doi: 10.1038/s41598-024-67331-z 39019997 PMC11255215

[16] Martland R Korman N Firth J Vancampfort D Thompson T Stubbs B. Can high-intensity interval training improve mental health outcomes in the general population and those with physical illnesses? A systematic review and meta-analysis. Br J Sports Med. (2022) 56:279–91. doi: 10.1136/bjsports-2021-103984 34531186

[17] Negeri ZF Levis B Sun Y He C Krishnan A Wu Y . Accuracy of the patient health questionnaire-9 for screening to detect major depression: updated systematic review and individual participant data meta-analysis. BMJ. (2021) 375:n2183. doi: 10.1136/bmj.n2183 34610915 PMC8491108

[18] Aktürk Z Hapfelmeier A Fomenko A Dümmler D Eck S Olm M . Generalized anxiety disorder 7-item (GAD-7) and 2-item (GAD-2) scales for detecting anxiety disorders in adults. Cochrane Database Syst Rev. (2025) 3:Cd015455. doi: 10.1002/14651858.CD015455 40130828 PMC11934853

[19] Krupa-Kotara K Markowski J Gdańska A Grajek M Działach E Szlachta G . Global self-esteem, body composition, and physical activity in Polish University students. Nutrients. (2023) 15:3907. doi: 10.3390/nu15183907 37764691 PMC10536466

[20] Buysse DJ Reynolds CF. 3rd, Monk TH, Berman SR, Kupfer DJ. The Pittsburgh sleep quality index: a new instrument for psychiatric practice and research. Psychiatry Res. (1989) 28:193–213. doi: 10.1016/0165-1781(89)90047-4 2748771

[21] Ware JE. Jr., Sherbourne CD. The MOS 36-item short-form health survey (SF-36) I Conceptual framework and item selection. Med Care. (1992) 30:473–83. doi: 10.1097/00005650-199206000-00002 1593914

[22] Tao Y Lu J Lv J Zhang L. Effects of high-intensity interval training on depressive symptoms: a systematic review and meta-analysis. J Psychosom Res. (2024) 180:111652. doi: 10.1016/j.jpsychores.2024.111652 38603999

[23] Zuo Z Zhang Z Li Y Zhang J Shi P. The effect of high-intensity interval training on inhibitory function in overweight female college students: the mediating role of body composition. BMC Psychol. (2025) 13:272. doi: 10.1186/s40359-025-02479-5 40108722 PMC11921596

[24] Gaweł E Hall B Siatkowski S Grabowska A Zwierzchowska A. The combined effects of high-intensity interval exercise training and dietary supplementation on reduction of body fat in adults with overweight and obesity: a systematic review. Nutrients. (2024) 16:355. doi: 10.3390/nu16030355 38337640 PMC10857230

[25] Yu P Zhu Z He J Gao B Chen Q Wu Y . Effects of high-intensity interval training, moderate-intensity continuous training, and guideline-based physical activity on cardiovascular metabolic markers, cognitive and motor function in elderly sedentary patients with type 2 diabetes (HIIT-DM): a protocol for a randomized controlled trial. Front Aging Neurosci. (2023) 15:1211990. doi: 10.3389/fnagi.2023.1211990 37649720 PMC10465302

[26] Poon ET Wongpipit W Li HY Wong SH Siu PM Kong AP . High-intensity interval training for cardiometabolic health in adults with metabolic syndrome: a systematic review and meta-analysis of randomised controlled trials. Br J Sports Med. (2024) 58:1267–84. doi: 10.1136/bjsports-2024-108481 39256000

[27] Zeng J Wang H. The impact of high-intensity exercise on patients with depression: a systematic review and meta-analysis of randomized controlled trials. Front Public Health. (2025) 13:1616925. doi: 10.3389/fpubh.2025.1616925 40880927 PMC12380541

[28] Schuch FB Vancampfort D. Physical activity, exercise, and mental disorders: it is time to move on. Trends Psychiatry Psychother. (2021) 43:177–84. doi: 10.47626/2237-6089-2021-0237 33890431 PMC8638711

[29] Scott AJ Webb TL Martyn-St James M Rowse G Weich S. Improving sleep quality leads to better mental health: a meta-analysis of randomised controlled trials. Sleep Med Rev. (2021) 60:101556. doi: 10.1016/j.smrv.2021.101556 34607184 PMC8651630

[30] Poon ET Siu PM Wongpipit W Gibala M Wong SH. Alternating high-intensity interval training and continuous training is efficacious in improving cardiometabolic health in obese middle-aged men. J Exerc Sci Fit. (2022) 20:40–7. doi: 10.1016/j.jesf.2021.11.003 34987589 PMC8689221

[31] Cicek G Ozcan O Akyol P Isik O Novak D Küçük H. The effect of aerobic and high-intensity interval training on plasma pentraxin 3 and lipid parameters in overweight and obese women. PeerJ. (2024) 12:e18123. doi: 10.7717/peerj.18123 39372725 PMC11451446

[32] Min L Wang D You Y Fu Y Ma X. Effects of high-intensity interval training on sleep: a systematic review and meta-analysis. Int J Environ Res Public Health. (2021) 18:10973. doi: 10.3390/ijerph182010973 34682718 PMC8535574

[33] Guo Z Cai J Wu Z Gong W. Effect of high-intensity interval training combined with fasting in the treatment of overweight and obese adults: a systematic review and meta-analysis. Int J Environ Res Public Health. (2022) 19:4638. doi: 10.3390/ijerph19084638 35457507 PMC9030367

[34] Hadjispyrou S Dinas PC Delitheos SM Koumprentziotis IA Chryssanthopoulos C Philippou A. The effect of high-intensity interval training on mitochondrial-associated indices in overweight and obese adults: a systematic review and meta-analysis. Front Biosci. (2023) 28:281. doi: 10.31083/j.fbl2811281 38062841

[35] Fu J Zhang W Xu X Mao X Wang L Cai M . The impact of 4-week high-intensity interval training on mental health and sleep quality in female college students with normal weight obesity: a randomized controlled trial. J Transl Med. (2025) 23:1234. doi: 10.1186/s12967-025-07276-7 41199311 PMC12590694

[36] Pedersen BK Saltin B. Exercise as medicine - evidence for prescribing exercise as therapy in 26 different chronic diseases. Scand J Med Sci Sports. (2015) 25(Suppl. 3):1–72. doi: 10.1111/sms.12581 26606383

[37] Sallis RE. Exercise is medicine and physicians need to prescribe it! Br J Sports Med. (2009) 43:3–4. doi: 10.1136/bjsm.2008.054825 18971243

[38] Warburton DE Nicol CW Bredin SS. Health benefits of physical activity: the evidence. CMAJ. (2006) 174:801–9. doi: 10.1503/cmaj.051351 16534088 PMC1402378

